# Generation of IL-23 Producing Dendritic Cells (DCs) by Airborne Fungi Regulates Fungal Pathogenicity via the Induction of T_H_-17 Responses

**DOI:** 10.1371/journal.pone.0012955

**Published:** 2010-09-23

**Authors:** Georgios Chamilos, Dipyaman Ganguly, Roberto Lande, Josh Gregorio, Stephan Meller, William E. Goldman, Michel Gilliet, Dimitrios P. Kontoyiannis

**Affiliations:** 1 Department of Immunology, The University of Texas M. D. Anderson Cancer Center, Houston, Texas, United States of America; 2 Department of Infectious Diseases, Infection Control and Employee Health, The University of Texas M. D. Anderson Cancer Center, Houston, Texas, United States of America; 3 Department of Melanoma Medical Oncology, The University of Texas M. D. Anderson Cancer Center, Houston, Texas, United States of America; 4 Department of Microbiology and Immunology, The University of North Carolina at Chapel Hill, Chapel Hill, North Carolina, United States of America; Massachusetts General Hospital, United States of America

## Abstract

Interleukin-17 (IL-17) producing T helper cells (T_H_-17) comprise a newly recognized T cell subset with an emerging role in adaptive immunity to a variety of fungi. Whether different airborne fungi trigger a common signaling pathway for T_H_-17 induction, and whether this ability is related to the inherent pathogenic behavior of each fungus is currently unknown. Here we show that, as opposed to primary pathogenic fungi (*Histoplasma capsulatum*), opportunistic fungal pathogens (*Aspergillus* and *Rhizopus*) trigger a common innate sensing pathway in human dendritic cells (DCs) that results in robust production of IL-23 and drives T_H_-17 responses. This response requires activation of *dectin-1* by the fungal cell wall polysaccharide b-glucan that is selectively exposed during the invasive growth of opportunistic fungi. Notably, unmasking of b-glucan in the cell wall of a mutant of *Histoplasma* not only abrogates the pathogenicity of this fungus, but also triggers the induction of IL-23 producing DCs. Thus, b-glucan exposure in the fungal cell wall is essential for the induction of IL-23/T_H_-17 axis and may represent a key factor that regulates protective immunity to opportunistic but not pathogenic fungi.

## Introduction

In recent years, opportunistic fungal pathogens have become major causes of life-threatening infections in an expanding population of severely immunocompromised individuals, as a result of the increasing use of transplantation, the development of immunosuppressive therapies for autoimmune and neoplastic diseases, and the AIDS pandemic [1], [2]. In these patients, *Aspergillus* is the predominant fungal pathogen [3], whereas Zygomycetes have emerged as the second most common cause of opportunistic mold infections [4], [5]. Infections caused by these fungi have a poor prognosis with mortality rates exceeding 90% upon dissemination, mainly because of their inherent resistance to existing antifungal agents [1]–[5]. Thus, there is a need for better understanding of the pathogenesis of opportunistic fungal infections in order to develop novel therapeutic strategies.

T helper (T_H_) cells play a crucial role in host defense against fungi through the secretion of distinct cytokine profiles [6], [7]. Traditionally, responses by interferon-γ (IFN-γ)–producing T_H_-1 cells are considered to confer protective immunity against fungi, whereas T_H_-2 responses mediated by IL-4 lead to increased susceptibility to fungal infections [6], [7]. More recently, a subset of T_H_ cells, called IL-17-producing T_H_ (T_H_-17) cells, have been identified and implicated in mucosal immunity against extracellular bacteria [8], [9] and fungi [10]-[17]. In particular, IL-17 receptor deficient mice are susceptible to disseminated [10] and oropharyngeal [11] candidiasis, whereas impaired IL-17 production has been associated with increased susceptibility to fungal pneumonia caused by *Pneumocystis* [12], *Cryptococcus* [13], and *Aspergillus* ([14]. In humans, patients with mutations in STAT3 have selective impairment of IL-17 producing T cells and are prone to infections with invasive fungal pathogens, including *Candida* and *Aspergillus* [15]. Furthermore, *Candida*-specific human memory CD4^+^ T cells belong to the T_H_-17 subset [16], and are significantly decreased in patients with chronic mucocutaneous candidiasis [17].

IL-23, a member of the IL-12 cytokine family, has a master role in regulating T_H_-17 development [8], [9], [18]. Dendritic cells (DCs) are the main source of IL-23, which is secreted as a heterodimer comprising of a p19 subunit and a p40 subunit shared with IL-12 [18]. Human T_H_-17 cells originate from CD161^+^ T-cell precursors that constitutively express IL-23R [19], and naïve CD4^+^ T cells differentiate *in vitro* into mature T_H_-17 cells in response to the combined activity of IL-23 and IL-1b in the presence of TGF-b [19]–[22].

In contrast to IL-23, IL-12 is associated with the differentiation of naïve CD4^+^ T cell into IFN-γ secreting T_H_-1 cells [8], [9], [18]. Much effort has focused on identifying microbial ligands and the receptors that drive the differentiation of DCs into producers of either IL-12 or IL-23 [8], [9], [23]. In fungi, activation of members of the Toll-like receptor (TLR) family, mainly TLR2 and TLR4, in DCs has been linked to the induction of IL-12 leading to protective T_H_-1 responses against *Candida* and *Aspergillus* [24], [25]. On the other hand, activation of *dectin-1*, a C-type lectin expressed on DCs that recognizes the polysaccharide b-glucan, triggers the induction of IL-23 through the syk-CARD-9 signaling pathway [26], and drives robust T_H_-17 response [26]. Interestingly, while the yeast form of *Candida albicans* induces IL-12, the invasive, tissue-infiltrating form of the fungus (hyphae) selectively induces IL-23 and drives T_H_-17 responses [26]. Therefore, it appears that tissue invasion by fungi triggers activation of the IL-23/T_H_-17 pathway that has a central role in the initiation of an acute inflammatory response via neutrophil recruitment and the induction of epithelial antimicrobial peptides [6], [7]. The important role of *dectin-1*-syk-CARD-9 signaling in induction of T_H_-17 responses and protective mucosal immunity against *Candida* has been convincingly shown both in mice [27], [28] and recently in humans [29], [30].

In view of the complexity and the structural differences in the fungal cell wall of fungi [31], [32], whether activation of *dectin-1* signaling is a major pathway for T_H_-17 induction in response to airborne opportunistic fungal pathogens that currently cause the majority of fungal infections in immunocompromised patients, and whether this ability is related to specific growth stages and/or the inherent pathogenic behavior of each fungus in currently unknown.

Here we studied human DC responses against the two predominant airborne opportunistic fungal pathogens, *Aspergillus* and *Rhizopus*, and the pathogenic fungus *Histoplasma*. Importantly, we found that opportunistic fungal pathogens (*Aspergillus* and *Rhizopus*) but not primary pathogenic fungi (*Histoplasma*) prime dendritic cells (DCs) to produce high levels of IL-23, which drives T_H_-17 responses. The induction of these IL-23 producing DCs depended on activation of *dectin-1* and is mediated by b-glucan exposed in the cell wall of the invasive form of opportunistic fungal pathogens. By contrast, the yeast form of the pathogenic fungus *Histoplasma*, which lacks cell wall exposure of b-glucan, failed to induce IL-23 producing DCs. Notably, unmasking of b-glucan in the cell wall of a mutant of *Histoplasma*, not only abrogated the pathogenicity of this fungus, but also triggered the induction of IL-23 producing DCs. Thus, b-glucan exposure in the cell wall of fungi is essential for the generation of IL-23 producing DCs and T_H_-17 immune responses and may represent a key factor that regulates protective immunity to opportunistic but not primary pathogenic fungi.

## Results

### The invasive stage of growth of opportunistic fungal pathogens but not that of primary pathogenic fungi drives the induction of IL-23 producing DCs

**Figure 1:**
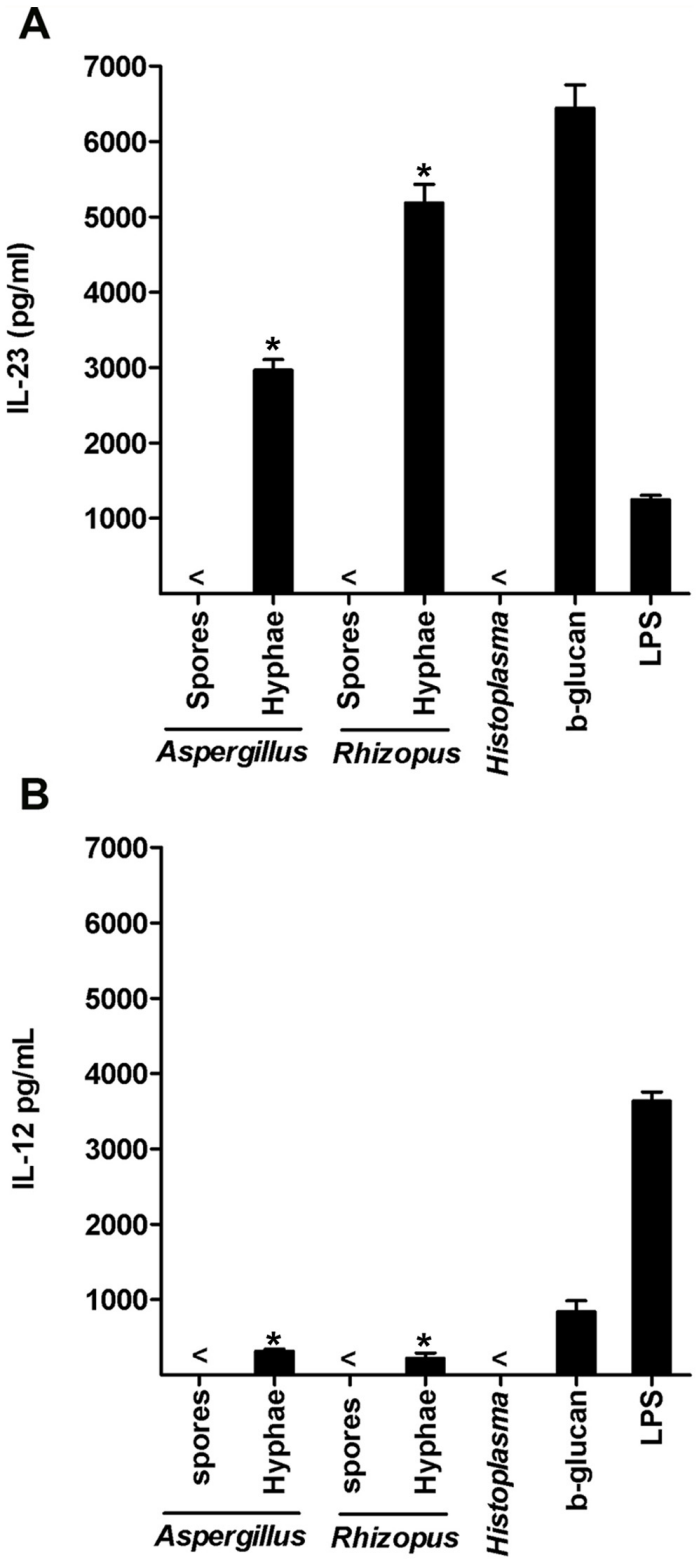
The invasive stage of growth of opportunistic fungal pathogens but not that of primary pathogenic fungi drives the induction of IL-23 producing DCs. IL-23 (A) and IL-12 (B) produced by human monocyte-derived DCs (1×10^6^ cells per ml) following overnight stimulation with purified b-glucan (curdlan, 100 µg/ml), LPS (100 ng/ml), resting (spores) or invasive (hyphae) stages of growth of each opportunistic fungal pathogen (*Aspergillus* and *Rhizopus*) and the invasive form (yeast) of the pathogenic fungus *Histoplasma*, at a 1∶1 ratio. The results are representative of 6 independent experiments. < indicates that the measured value was below the detection limit of the assay (<20 pg/ml). Error bars represent SD. *, *P*<0.001, paired Student's *t* test.

To test the ability of airborne fungi that cause human infections to drive DC-mediated T_H_-17 responses, we used *Aspergillus* and *Rhizopus as* model opportunistic fungal pathogens and *Histoplasma*, a pathogenic fungus that causes infections in immunocompetent individuals [6]. Previous studies suggested that DCs are capable of discriminating between the different stages of fungal growth (spores vs. hyphae) to prime the development of T_H_-1 and T_H_-2 responses [24], [25]. We therefore tested if the induction of T_H_-17 responses is also dependent on specific stage of growth of fungi by human DCs. Human DCs were infected with invasive stage (hyphae) or the resting stage (spores) of *Aspergillus* or *Rhizopus*, and cytokine secretion was analyzed after overnight culture. We found that DC infected with the invasive stage but not the resting stage of both fungi secreted high amounts of IL-23 (Figure 1A) and only low amounts of IL-12 (Figure 1B). Importantly, IL-23 production by DCs has been recognized to be a key driver of T_H_-17 responses [8], [9], [19], [23]. IL-23 producing DCs induced by the invasive stage of *Aspergillus* or *Rhizopus* also secreted high levels of IL-1, IL-6, and TNF-a (Figure S1).

Next, we evaluated DCs infected with the pathogenic fungus *Histoplasma*, one of the few fungi that cause invasive infections in immunocompetent individuals [6], [32]. *Histoplasma* is a dimorphic fungus that adapts a particular stage of growth (yeast) at mammalian temperatures [6], [32]. This growth adaptation is considered a virulence trait of *Histoplasma* that allows the fungus to escape immune recognition and establish persistent infection within host macrophages [32]. Interestingly, in contrast to opportunistic fungi, we found that the yeast form of *Histoplasma* failed to elicit IL-23 producing DCs (Figure 1A). Together, these data demonstrate that the invasive form of opportunistic fungal pathogens *Aspergillus* or *Rhizopus* but not of the pathogenic fungus *Histoplasma* drives the induction of IL-23 producing DCs.

### The invasive form of opportunistic fungal pathogens but not that of primary pathogenic fungi exposes b-glucan in the cell wall surface

**Figure 2:**
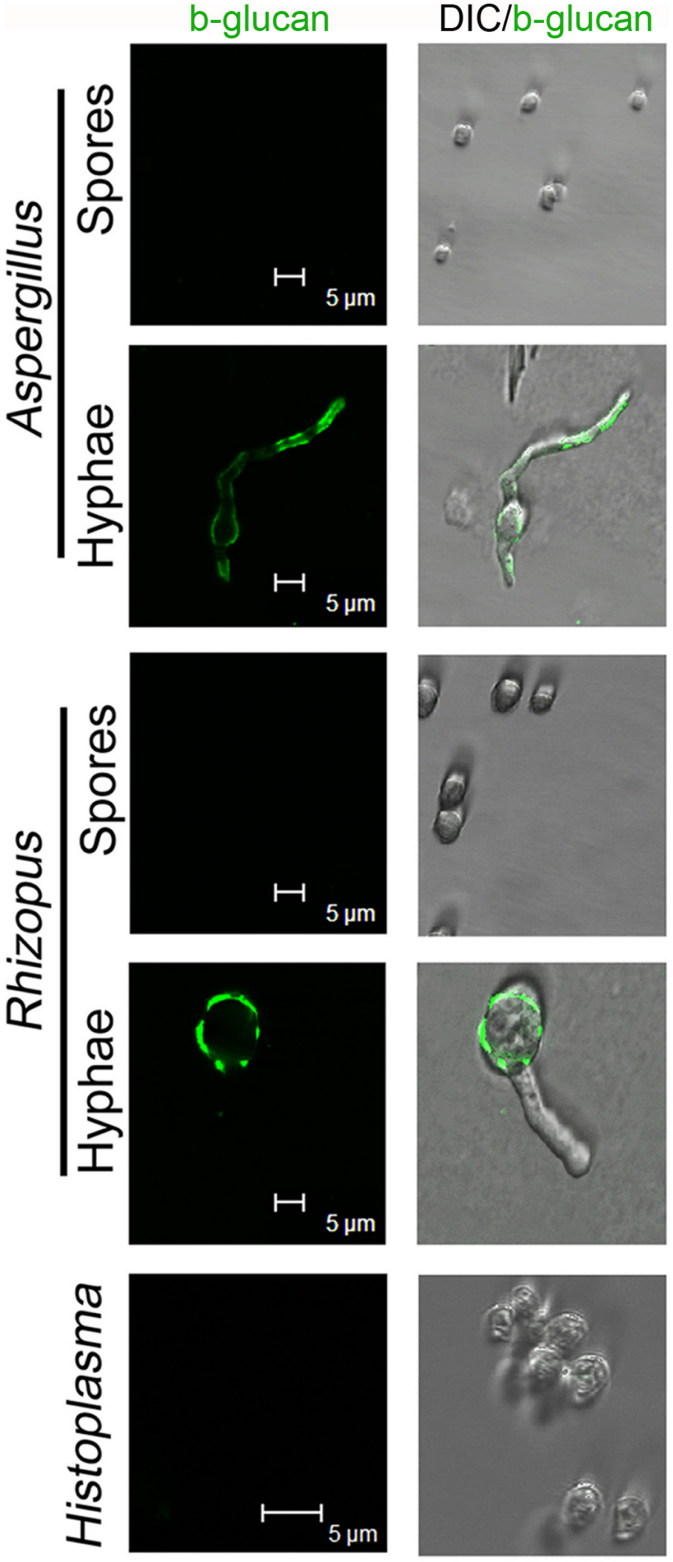
The invasive form of opportunistic fungal pathogens but not that of pathogenic fungi exposes b-glucan in the cell wall surface. Representative confocal microscopy images of b-glucan surface staining of resting (spores) and invasive (hyphae) stages of growth of the opportunistic fungi *Aspergillus* or *Rhizopus* and the invasive (yeast) form of the pathogenic fungus *Histoplasma*. Live fungal cells were fixed with 1% paraformaldehyde and stained with a mouse b-(1–3)-glucan-specific monoclonal antibody followed by staining with a secondary goat anti-mouse Alexa488 antibody (green). The antibody failed to detect surface b-glucan on the spores of opportunistic fungi and in the invasive form of *Histoplasma* but detected high amounts of the polysaccharide in germinating hyphae of both *Aspergillus* and *Rhizopus* (left panels). The overlay images (DIC/b-glucan immunostaining; right panels) indicate the pattern of surface b-glucan staining in the different stages of growth of fungi. Data shown are representative of 3 independent experiments.

b-glucan has been shown to be a key driver of IL-23 production in DCs [23], [26]. Previous studies have shown that *Aspergillus* exposes b-glucan on the cell wall surface during the invasive stage of growth (hyphae) [33]–[35]. Thus, we sought to determine whether like *Aspergillus* also *Rhizopus* but not *Histoplasma* exposes b-glucan in the cell wall of the invasive stage of fungal growth. Immunofluorescene studies using a b-glucan specific monoclonal antibody confirmed the exposure of b-glucan in hyphae of *Aspergillus* (Figure 2). Interestingly, abundant staining of b-glucan was also found on the surface of hyphae of *Rhizopus* (Figure 2), which is in contrast to previous reports that failed to identify b-glucan on the cell wall of *Rhizopus* and instead suggested that chitin and chitosan comprise the main polysaccharides of this fungus [36]. Importantly, there was no evidence of b-glucan staining in the spores of both opportunistic fungal pathogens and in the yeast form of the pathogenic fungus *Histoplasma* (Figure 2). These data indicate that b-glucan exposure selectively occurs in the invasive stage of growth of opportunistic fungal pathogens but not in primary pathogenic fungi, a finding that correlates with the ability of these fungi to induce IL-23 producing DC.

### Induction of IL-23 producing DCs by opportunistic fungal pathogens is dectin-1 dependent and is mediated by b-glucan

**Figure 3:**
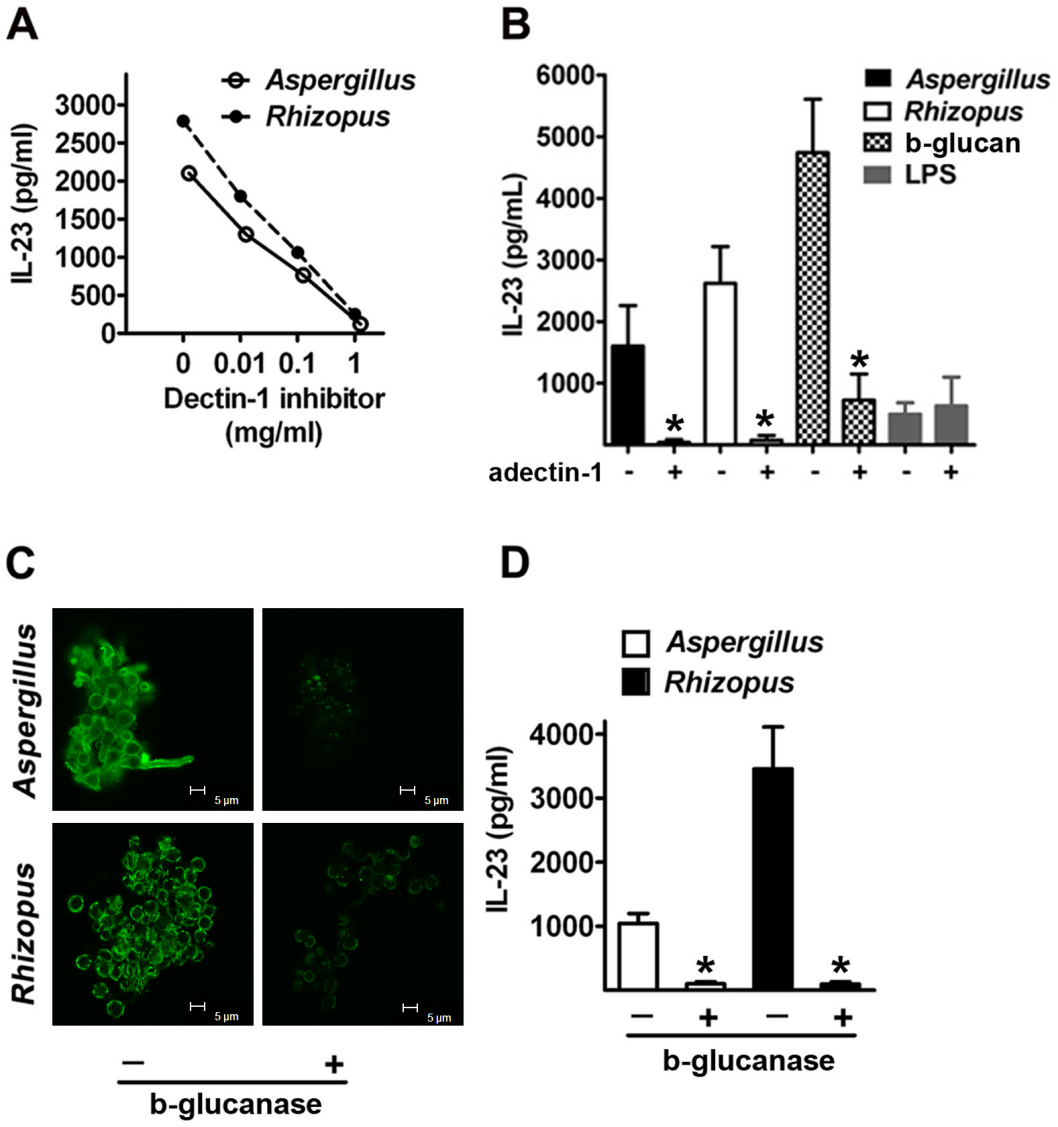
Induction of IL-23 producing DCs by opportunistic fungal pathogens is dectin-1 dependent and is mediated by b-glucan. (A) IL-23 production by human monocyte-derived DCs pre incubated for 1 h with increasing concentrations of the *dectin-1* inhibitor laminarin (0, 0.01, 0.1, and 1 mg/ml) and subsequently stimulated with hyphae of either *Aspergillus* (open cycles) or *Rhizopus* (closed cycles), at a 1∶1 ratio. (B) IL-23 production by DCs stimulated with hyphae of either *Aspergillus* (black bars) or *Rhizopus* (white bars), or b-glucan (scattered bars) or LPS (gray bars) alone or following 1 h pre incubation with an anti-dectin-1 blocking antibody (10 µg/ml). Data are expressed as mean ± SEM values for DCs derived from three different donors. (C) Representative confocal microscopy images of b-glucan surface staining in hyphae of *Aspergillus* or *Rhizopus* untreated or following overnight enzymatic digestion with b-glucanase (10 U/ml). (D) IL-23 production by DCs stimulated with hyphae of *Aspergillus* (white bars) or *Rhizopus* (black bars) untreated or following overnight enzymatic digestion with b-glucanase. Data shown are representative of 3 independent experiments. Error bars represent SD. *, *P*<0.001, paired Student's *t* test.

Next, we sought to demonstrate that the selective exposure of b-glucan in the hyphae of opportunistic fungal pathogens drives the induction of IL-23 producing DCs. We used laminarin, a competitive inhibitor of b-glucan for its binding to *dectin-1*, the receptor for b-glucan expressed on DCs [27], [37]. Increasing concentrations of laminarin abrogated the induction of IL-23 production by DCs infected with hyphae of *Aspergillus* or *Rhizopus* (Figure 3A). As a control, we stimulated DCs with the TLR4 agonist LPS, and showed that laminarin had no effect on the production of IL-23 by DCs (Figure S2). Furthermore, pre-incubation of DCs with a neutralizing anti-*dectin-1* antibody resulted in complete inhibition of IL-23 production in DCs infected with hyphae of both opportunistic fungi, while it had no effect on IL-23 production by DCs stimulated with LPS (Figure 3B).

Because b-glucan is not the only ligand of *dectin-1* [37], [38], we further investigated the role of b-glucan surface exposure by specific enzymatic digestion of b-glucan in the fungal cell walls using b-glucanase. The ability of b-glucanase to digest b-glucan in the cell wall of *hyphae of Aspergillus* and *Rhizopus* was confirmed by immunofluorescene studies (Figure 3C). DCs infected with b-glucanase-treated hyphae were unable to produce any IL-23 (Figure 3D), indicating that hyphae of opportunistic fungal pathogens induce IL-23 producing DCs through b-glucan surface exposure.

### Enforced b-glucan surface exposure in the non-pathogenic Histoplasma Δags1 mutant drives IL-23 producing DCs

In contrast to opportunistic fungal pathogens, in *Histoplasma* cell wall immunostimulatory b-glucans are covered in the yeast (infectious) form of the fungus by a layer of a-(1–3)-glucan [32], [39]. Concealing of b-glucan by non-stimulatory a-(1–3)-glucan molecules is regarded as a virulence mechanism, because it allows *Histoplasma* to avoid immune recognition and survive within the host cells. As a proof of principle, b-glucan surface exposure in the *Δags1* mutant of *Histoplasma capsulatum* that lacks a-(1–3)-glucan, renders it non-pathogenic in mice and results in increased release of TNFa in infected mouse macrophages through *dectin-1* activation [39].

**Figure 4:**
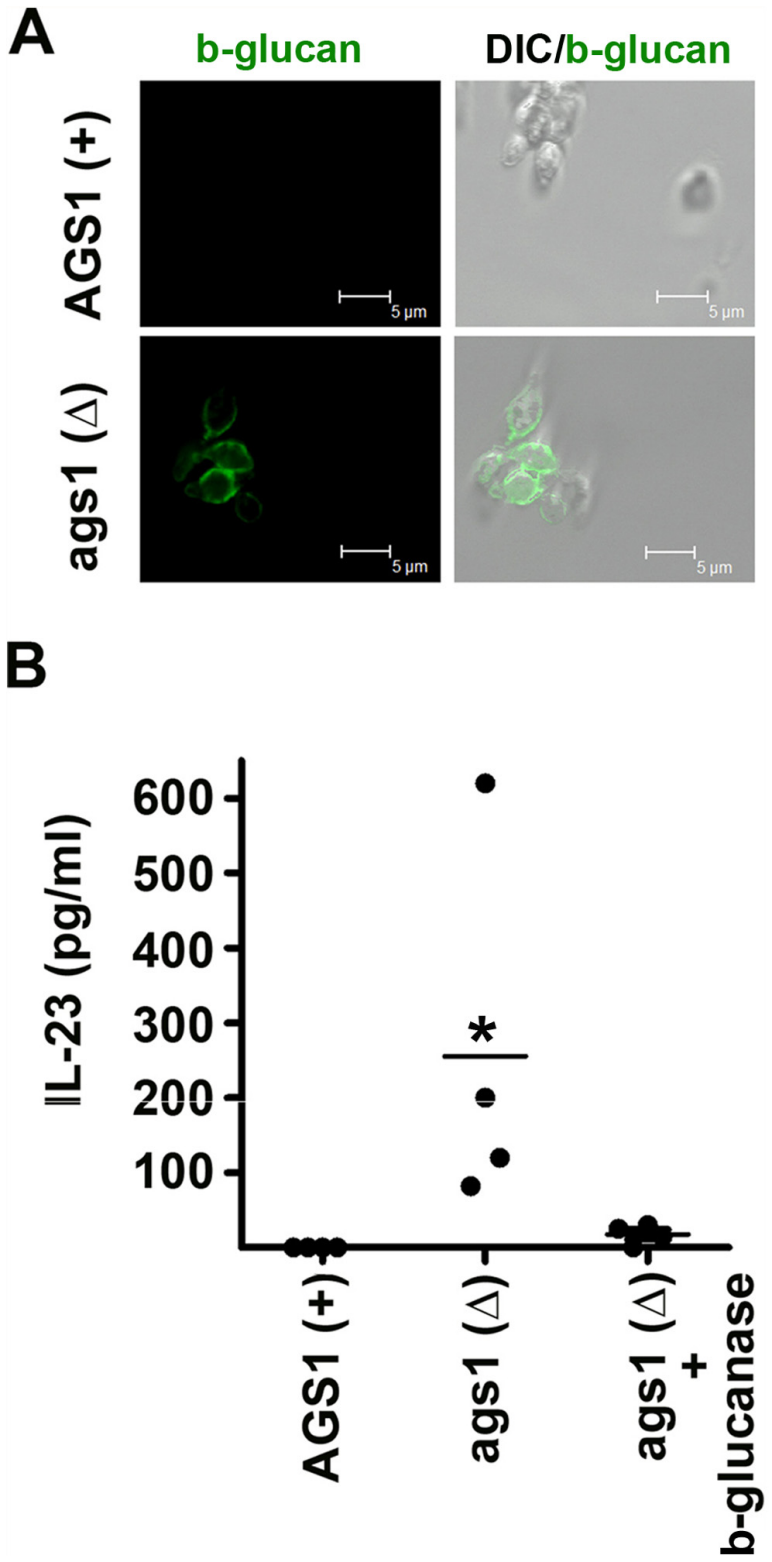
Enforced b-glucan surface exposure in the non-pathogenic Histoplasma Δags1 mutant drives IL-23 producing DCs. (A) Representative confocal microscopy images of b-glucan surface staining in yeast cells in the *Δags1* mutant of *Histoplasma* and the isogenic complementary strain *Ags1* + (left panel). Overlay images indicate the pattern of surface exposure on yeast cell surface (DIC/b-glucan immunostaining; right panel). (B) IL-23 production by DCs stimulated with the isogenic complementary strain *Ags1+* of *Histoplasma* and the *Δags1* mutant untreated or following overnight enzymatic digestion with b-glucanase (10 U/ml). IL-23 was selectively produced following stimulation of DCs with *Δags1* mutant. Data are expressed as mean ± SEM values of four independent experiments. *, *P*<0.001, paired Student's *t* test.

We hypothesized that similar to the induction of IL-23 release by b-glucan exposure in the hyphae of opportunistic fungal pathogens, unmasking of b-glucan in the *Δags1* mutant of *Histoplasma* should trigger the release of IL-23 in human DCs. We initially confirmed by immunofluorescene studies that the yeast form of the *Δags1* mutant of *Histoplasma* had surface exposure of b-glucan as opposite to its isogenic complementary *Ags1+* strain (Figure 4A). Importantly, infection of human DCs with the yeast form of the *Δags1* mutant of *Histoplasma* resulted in the production of IL-23 (Figure 4B); in contrast, there was no IL-23 production in DCs infected with the isogenic complementary *Ags1+* strain of *Histoplasma* (Figure 4B). Additionally, IL-23 production by human DCs infected with the *Δags1* mutant was inhibited following blocking of *dectin-1* receptor or enzymatic digestion of b-glucan (Figure 4B). These results further support the role of b-glucan in driving IL-23 producing DCs and indicate that the exposure of cell wall b-glucans may restrict the pathogenic potential of fungi via the induction of the IL-23-T_H_-17 response pathway.

### IL-23 producing DCs induced by opportunistic fungal pathogens drive T_H_-17 responses

**Figure 5:**
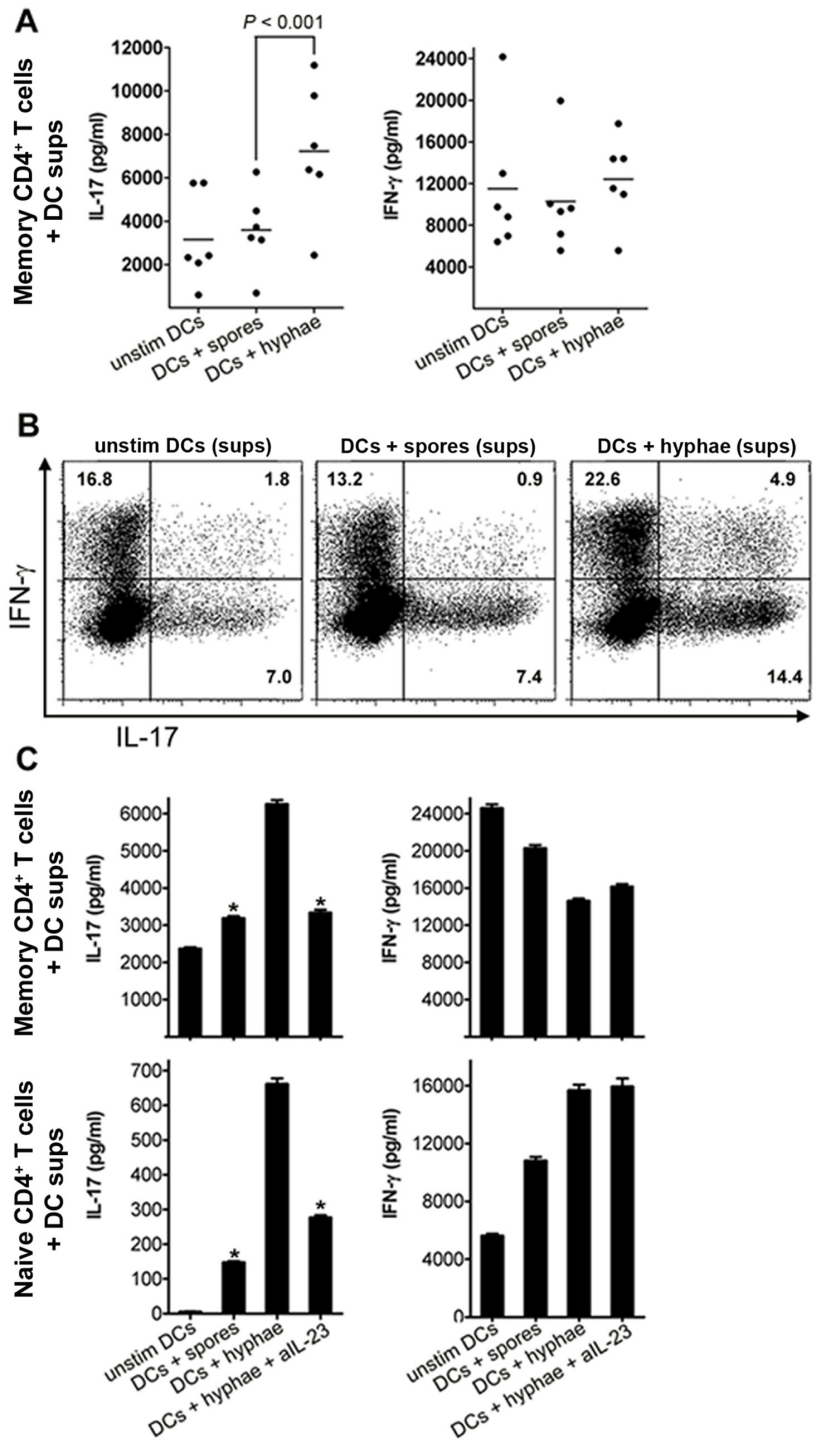
IL-23 producing DCs induced by opportunistic fungi drive Th17 responses. (A) IL-17 and IFN-γ production determined by ELISA in memory T cell cultures primed for 5 d in plates coated with anti-CD3 and anti-CD28, in the presence of supernatants (sups) of unstimulated DCs, or sups of DCs stimulated with either *Aspergillus* spores, or hyphae (above plots), and then re-stimulated for 24 h with anti-CD3 and anti-CD28. Data are expressed as mean ± SEM values for DCs derived from six different donors. (B) Intracellular cytokine staining for IL-17 and IFN-γ in memory CD4^+^ T cells primed and expanded as described in (A), and re-stimulated for 5 h with PMA and ionomycin. (C) ELISA of IL-17 and IFN-γ in 24-hour culture supernatants (sups) of naive and memory CD4^+^ T cells primed for 5 d in plates coated with anti-CD3 and anti-CD28, in the presence of sups of unstimulated DCs or *Aspergillus* spores-stimulated DCs or *Aspergillus*-hyphae stimulated DCs alone or in the presence of neutralizing anti-IL-23 antibodies. Data shown are representative of 5 independent experiments. Error bars represent SD. *, *P*<0.001, paired Student's *t* test.

Because IL-23 producing DCs represent the key drivers of T_H_-17 responses (8, 9, 19, 23), we next sought to determine whether human DCs stimulated with opportunistic fungal pathogens are capable of driving T_H_-17 responses. Purified human naïve and memory CD4^+^ T cells were activated polyclonally in the presence of supernatants of DCs previously stimulated with hyphae or spores of *Aspergillus*. DCs stimulated with hyphae but not spores of *Aspergillus* induced a predominant increase in IL-17 production versus IFN-γ, indicating that the invasive form of this fungus can trigger DC-mediated T_H_-17 responses (Figure 5A). This finding was confirmed by intracellular cytokine staining of primed CD4^+^ T cells (Figure 5B), as there was a significant increase in IL-17^+^/IFN-γ^−^ single positive CD4^+^ T cells (mean increase from 4.6% to 9,7%, n = 5 experiments; *P* = 0.004) and IL-17^+^/IFN-γ^+^ double positive CD4+ T cells (mean increase from 1,1% to 9,7%; n = 5 experiments; *P* = 0.002), (Figure S4).

T_H_-17 responses by human DCs are typically driven by IL-23, but may also require, in particular at the initial stages of differentiation other pro inflammatory cytokines, including IL1b or IL1b plus IL-6 [19]–[23]. We found that IL-23 producing DCs also secreted large amounts of IL-1b, which was dependent on activation of *dectin-1* as it was completely abolished in the presence of *dectin-1* neutralizing antibody (Figure S3A). In addition, IL-6 was also produced by IL-23 producing DCs, although its secretion was only partially dependent on activation *of dectin-1* (Figure S3B). Notably, there was no significant reduction in IL-6 production by DCs stimulated with curdlan or fungal hyphae following the addition of polymyxin-B (PMX-B) (Figure S5), thus excluding LPS contamination in DC cultures.

The addition of neutralizing antibodies against IL-23 inhibited IL-17 production by naïve and memory CD4^+^ T cells polarized with *Aspergillus*-stimulated DCs close to the baseline levels (Figure 5C). Importantly, neutralizing antibodies against IL-23 did not affect IFN-γ production by CD4^+^ T cells polarized with *Aspergillus*-stimulated DCs (Figure 5C). Therefore, IL-23 producing DCs induced by hyphae of opportunistic fungi drive T_H_-17 responses.

## Discussion

The emergence of life-threatening infections caused by airborne opportunistic fungal pathogens in an expanding population of non-neutropenic patients with defects in T-cell function, such as transplant recipients, patients with autoimmune diseases receiving biologic therapies or corticosteroids, and individuals with AIDS [1], [2], illustrates the pivotal role of adaptive immunity in antifungal host defense.

Recent studies in mice demonstrate that the newly identified T_H_-17/IL-23 axis regulates epithelial host defense against a growing list of fungi [10]–[14]. In view of the considerable differences in generation and function of T_H_-17 cells between mice and humans [8], [9], understanding the regulatory mechanisms for induction of antifungal T_H_-17 responses in humans is important for development of targeted therapeutic strategies. Although purified b-glucan acts as a potent adjuvant for the induction of IL-23 producing DCs and T_H_-17 priming [26], the structural complexity and differential cellular composition of various fungi makes it difficult to predict whether, which, and how common molecular patterns in cell wall of different fungi link innate sensing by DCs with T_H_-17 pathway induction. Herein, we identified a common innate sensing pathway in human DCs that is triggered by opportunistic fungi. We found that this pathway was dependent on activation of *dectin-1* signaling by b-glucan exposure in the hyphae of opportunistic fungal pathogens, and resulted in robust induction of IL-23 producing DCs that primed T_H_-17 antifungal responses. Importantly, we also demonstrated that pathogenic fungi evade T_H_-17 induction by concealing immunostimulatory b-glucans with other polysaccharide layers and thus avoiding activation of *dectin-1* signaling.

Our initial studies identified that the signature cytokine of the T_H_-17 pathway, IL-23, was selectively produced by human DCs infected with hyphae of *Aspergillus* or *Rhizopus*, by not by DCs infected with dormant spores of opportunistic fungi or the yeast form of the pathogenic fungus *Histoplasma*. Importantly, we found that generation of these IL-23 producing DCs correlated with selective b-glucan surface exposure in the hyphae of *Aspergillus* and *Rhizopus*. This was an unexpected finding based on biochemical studies that failed to identify b-glucan in the cell wall of *Rhizopus* (36), and clinical evidence of lack of detectable amounts of b-glucan release in human infections caused by this fungus [4], [5]. Our results corroborate with recent studies that identified b-glucan synthetase, the enzyme responsible for synthesis of b-glucan, in *Rhizopus* [40]. Next, by using specific *dectin-1* inhibitors and b-glucan enzymatic digestion, we confirmed that induction of IL-23 producing DCs is dependent on activation of *dectin-1* signaling and is mediated by b-glucan exposure in the hyphae of opportunistic fungi. Importantly, surface exposure of b-glucan in the *Δags1* mutant of Histoplasma that lacks the upper layer of a-glucan, not only abrogated its virulence, but also triggered the induction of IL-23 producing DCs via activation of *dectin-1* signaling.

Our study is the first to explore the interaction of DCs with fungi of the class Zygomycetes and the regulatory mechanisms of T_H_-17 induction against airborne opportunistic fungi in humans. In agreement to our findings, a recent study reported that dormant spores of *Aspergillus* were immunologically inert and did not induce DC or alveolar macrophage activation, because of the presence of a surface ‘rodlet layer’, composed of the hydrophobic RodA protein that masks underlying immunostimulatory molecules [41].

Previous studies suggested that coordinated activation of TLR-2 and TLR-4 by *Aspergillus* spores triggers a T_H_-1 protective response, whereas *Aspergillus* hyphae trigger TLR-2 activation and a T_H_-2 response [6], [24]. Nonetheless, studies using different TLR and MyD88 knockout mice failed to convincingly show increased susceptibility to *Aspergillus* infection in the absence of immunosupression [7]. In contrast, *dectin-1* knockout mice, display increased susceptibility to invasive aspergillosis without the need for administration of immunosuppressive agents, significantly attenuated proinflammatory response and decreased IL-17 production in the lung [14]. Notably, in agreement to our finding, macrophages from *dectin-1* knockout mice fail to produce proinflammatory cytokines in response to *Aspergillus* infection [14]. Nonetheless, we cannot preclude that other immunostimulatory molecules on the fungal cell wall are selectively exposed during invasive fungal growth to trigger PRRs in human DCs [42]–[44]. Likewise, collaborative activation of *dectin-1* with surface TLRs and/or other PRR may be implicated in innate sensing of different opportunistic fungi [45], [46].

The induction of IL-23 producing DCs by different microbial ligands is a key driver of T_H_-17 responses [8], [9], [19], [23]. The ability of *dectin-1* when compared to other PRRs for preferentially induction of IL-23 over IL-12, may be related to activation of a noncanonical NF-kappaB signaling pathway mediated by the serine-threonine kinase Raf-1 [47]. Indeed, supernatants from IL-23 producing DCs generated by stimulation with *Aspergillus* hyphae or b-glucan induced the production of IL-17 in naive human CD4^+^ T cells and a robust expansion of IL-17 producing memory human CD4^+^ T cells stimulated polyclonally. Notably, IL-17 production was significantly reduced by an antibody neutralizing IL-23, as also suggested by other studies [19], [21]–[23].

Overall, our studies demonstrate the important role of *dectin-1* signaling as a major innate sensing pathway for induction on T_H_-17 antifungal responses in humans. It is plausible that dysregulation of this pathway in patients with underlying immunodeficiency leads to unrestricted growth of opportunistic fungi and the development of life-threatening infections. Therefore, targeting this pathway for immunotherapy is an appealing strategy for prevention and treatment of patients at increased risk for development of opportunistic fungal infections. Future studies on the function of *dectin-1*/T_H_-17 axis in immunocompromised individuals might also provide a tool for risk stratification for development of fungal infections.

## Materials and Methods

### Reagents

Highly purified *Escherichia coli* LPS, laminarin from *Laminaria digitata*, and b-1,3-D-glucanase from *Helix pomatia* were obtained from Sigma-Aldrich (St. Louis, MO). Purified particulate b-glucan (curdlan) was from Waiko (Tokyo, Japan). b-(1-3)-glucan-specific monoclonal antibody was from Biosupplies (Parkville, Australia). Blocking monoclonal antibodies for *dectin-1* (MAB1859, clone 259931; 10 µg/ml) and appropriate isotype control antibodies were from R&D Biosystems. Neutralizing antibodies for the IL-23 p19 subunit (AF1716; 10 µg/ml), and IL-4 (34019; 10 µg/ml) were from R&D Biosystems. Neutralizing antibodies for IFN-γ (B27; 10 µg/ml) were from BD Biosciences.

### Microorganisms and culture conditions

The *Aspergillus fumigatus* strain Af293 and the *Rhizopus oryzae* clinical isolate 557969 were grown on YAG-chloramphenicol agar plates for 3 days at 37°C. Fungal spores in the presence of sterile 0.1% Tween 20 in PBS were harvested by gentle shaking, washed, filtered, counted by a hemacytometer, and suspended at a concentration of 10^8^ spores/ml. Germinating hyphae of *Aspergillus* and *Rhizopus* were obtained following growth in liquid RPMI 1640 media in a shaking incubator (220 rpm, 37°C), for 12 h and 8 h, respectively. Typically, >80% of conidia of each fungus were visibly swollen after 5 h.

Enzymatic digestion of b-glucan in the hyphae of *Aspergillus* and *Rhizopus* was performed by using b-1-3-D-glucanase from *Helix pomatia* (Sigma). Hyphae of both fungi were incubated overnight in a shaking bath with 10 U/ml of b-glucanase at a temperature of 55°C and pH 5, 0. Inactivation of enzyme was achieved by 10 min incubation at 100°C followed by three washes in PBS. Verification of b-glucan digestion was performed by immunostaining with a b-glucan monoclonal antibody.

*H. capsulatum Δags1* mutant and its isogenic complementary strain *AGS1* (+) were derived from the clinical isolate G186A (ATCC 26029) and have been described in ref 39. *Histoplasma* yeasts were grown at 37°C with 95% air/5% CO_2_ in HMM supplemented with 100 µg/ml uracil. Dispersed *Histoplasma* yeasts were obtained by growth of liquid cultures to late exponential phase, removal of large yeast clumps by low-speed centrifugation (60 s at 100×*g*), three washes with Ham's F-12 (Invitrogen, Carlsbad, CA), and counting with a hemacytometer.

Inactivation of fungi was done by heat exposure (30 min, 65 C) or exposure to 1% paraformaldehyde (4°C, overnight). In initial DC stimulation experiments, amphotericin B (1 µg/ml) was added to the cell cultures immediate after infection with fungi to prevent germination or restrict overgrowth of germinating hyphae. Because there were no appreciable differences in cytokine production as compared to infection of DCs with inactivated fungi, all following studies were performed with inactivated fungal cells.

### Generation of monocyte-derived DCs

This study was approved by the Institutional Review Board for human research at the M. D. Anderson Cancer Center in Houston. Separation of peripheral blood mononuclear cells (PBMCs) was performed by density gradient centrifugation using ficoll hypaque (Amersham Pharmacia Biotech, Uppsala, Sweden) from healthy donor blood obtained from the Gulf Coast Regional Blood Center, Houston, Texas. CD14^+^ cells were enriched from the PBMCs using CD14 microbeads and columns (Miltenyi Biotec, Auburn, CA). In order to generate DCs, CD14^+^ cells were cultured in the presence of IL-4 (100 ng/mL; R&D Systems, Minneapolis, MN) and granulocyte-macrophage colony-stimulating factor (GM-CSF; 100 ng/mL R&D Systems, Minneapolis, MN) for 5 days at 2×10^6^ cells/ml in RPMI 1640 supplemented with2 mM l-glutamine, 1 mM sodium pyruvate, 10 mM HEPES, 100 U/ml penicillin, 100 µg/ml streptomycin, and 10% FCS (v/v). On day 5 the cells were 70 to 90% CD1a^+^ and 95% CD14^−^. Then the culture medium containing IL-4 and GM-CSF was replaced with culture medium alone 2 h before infection with fungi or other treatments.

### Purification of naive and memory CD4^+^ T lymphocytes from adult blood

Untouched naïve and memory CD4^+^ T lymphocytes were purified from PBMCs by immunomagnetic depletion with the naïve CD4^+^ T Cell Isolation Kit II and memory CD4^+^ T Cell Isolation Kit (Miltenyi Biotec), respectively. Naive CD4^+^ T cells (CD3^+^CD4^+^CD45RA^+^CD45RO^−^) typically had a purity of over 95%, as evidenced by flow cytometry. For some experiments, peripheral blood CD4^+^ T cells were isolated with the CD4^+^ T Cell Isolation Kit II (Miltenyi Biotec), followed by staining with APC-CD45RO^+^, FITC-CD45RA+, PE-Cy5-CD4^+^, and PE-labeled lineage mixture antibodies against CD8, CD14, CD16, CD19, CD56, CD11c, γδ-TCR, CD11c, CD25, and BDCA-2, and were sorted into two fractions of CD4^+^ **CD45RO^+^**CD45RA^−^ and CD4^+^ **CD45RA^+^**CD45RO^−^ with cell purity over 99%, on a FACSAria (BD Bioscience).

### Stimulation of monocyte-derived DCs

Monocyte-derived DCs were seeded at a final concentration of 10^6^ cells per ml in RPMI culture medium in flat-bottomed 24-well plates (Falcon). Spores or hyphae of each opportunistic fungus or yeast cells of each strain of *Histoplasma* were counted, and added to DC cultures at a final concentration of 10^6^ cells per ml (1∶1 E:T ratio). In order to normalize for the amount of spores and hyphae that were co-cultured with DCs and minimize the effects of the increased fungal biomass of hyphae, we used early germinating hyphae (fig. 2) instead of mature, branching hyphae. In pilot experiments, increasing the amount of dormant spores to 1∶10 or 1∶ 20 didn't have a significant effect on the type and amount of pro-inflammatory cytokines produced by DCs. Stimulation of DCs was also induced in flat-bottomed 24-well plates by adding purified b-glucan (curdlan; 100 µg/ml), or LPS (100 ng/ml). For receptor blocking experiments DCs were pre incubated with blocking antibodies for *dectin-1*, or the corresponding isotype control antibody for 1 h before treatment with fungi or other stimuli.

### T cell stimulation

Naïve and memory CD4^+^ T cell subsets were cultured in flat-bottomed 96-well plates (Falcon) in Yssel's medium supplemented with 2 mM l-glutamine (GIBCO-Invitrogen), 1 mM sodium pyruvate (GIBCO-Invitrogen), 10 mM HEPES (GIBCO-Invitrogen),100 U/ml penicillin, 100 µg/ml streptomycin (GIBCO-Invitrogen), and 10% FCS (v/v; GIBCO-Invitrogen), at a density of 5×10^4^ cell per well, and stimulated with plate-bound anti-CD3 (10 µg/ml) and soluble anti-CD28 (1 µg/ml) in the presence of supernatants from unstimulated or variously stimulated monocyte-derived DCs. For some experiments with naïve CD4^+^ T cells, anti-IFN-γ and anti-IL-4 were added to the cultures at a concentration of 10 µg/ml. After 5–6 days cells were collected and washed extensively and their viability was determined by trypan blue exclusion. Cells (1×10^6^ cells/ml) were restimulated with PMA (50 ng/ml; Sigma) and Ionomycin (500 ng/ml; Sigma) for 6 h (for flow cytometry intracellular staining) or in the presence of anti-CD3 and anti-CD28 for 24 h (for ELISA).

### Analysis of cytokine production

Supernatants of stimulated DCs or T cells were collected after overnight culture. Cytokine levels in the supernatants were determined by using ELISA kits for human IL-1b, IL-6, IL-12, IL-17, IFN-γ (R&D systems), and IL-23 ELISA (eBioscience) according to the manufacturer's instructions. Cells producing IFN-γ and IL-17 were analyzed by intracellular cytokine staining after the addition of GolgiStop (10 µg/ml; BD Biosciences) during the final 4 h of restimulation. Cells were made permeable with Cytofix/Cytoperm reagents (BD Biosciences). Cells were stained with FITC-anti-IFN-γ (4S.B3; BD Pharmingen) and PE-anti-IL-17 (eBio 64DEC17; eBioscience) and washed and then were analyzed by flow cytometry (FACSCalibur; BD Biosciences).

### Confocal imaging studies

Live spores or hyphae of opportunistic fungi and yeast cells of *Histoplasma* were fixed with 4% paraformaldehyde, pelleted in propylene tubes, washed twice with PBS, blocked for 30 min in PBS plus 1% goat serum, incubated for 1 h with a mouse monoclonal antibody to linear-(1,3)-b-glucan (Biosupplies; 1 µg/ml), washed twice in PBS plus 1% goat serum and stained by a secondary AlexaFluor 488 goat anti-mouse Ab (Molecular probes). Images of PFA-fixed fungal cells were acquired using a Leica SP2 RS laser-scanning confocal microscope with an oil-immersion objective (Leica ×63/1.4 numerical aperture) using identical gain settings at room temperature. The AlexaFluor 488 goat anti-mouse Ab used as flurochrome was excited with an Argon Laser. Images were collected using LCS V2.61 software (Leica Microsystems, GmbH) and processed with Adobe Photoshop CS2.

#### Statistics

A standard two-tailed t-test or a t-test with Welch's correction was applied for statistical analysis by using the GraphPad Prism software. *P* values of 0.05 or less were considered significant.

## Supporting Information

Figure S1 TH-17 polarizing cytokines are preferentially induced by fungal hyphae in human DCs. IL-1b (A) IL-6 (B) and TNF-a produced by human monocyte-derived DCs (1×10^6^ cells per ml) following overnight stimulation with purified b-glucan (curdlan, 100 µg/ml), resting (spores) or invasive (hyphae) stages of growth of each opportunistic fungus (Aspergillus and Rhizopus) at a 1∶1 ratio. Data are expressed as mean ± SEM values for DCs derived from three different donors. *, P<0.001, paired Student's t test.
(1.08 MB TIF)

Figure S2 Dectin-1 inhibitor laminarin has no effect on DC activation by TLR ligands. IL-23 production by human monocyte-derived DCs pre incubated for 1 h with increasing concentrations of the dectin-1 inhibitor laminarin (0, 0.01, 0.1, and 1 mg/ml) and subsequently stimulated with LPS (100 ng/ml). Data shown are representative of 2 independent experiments.
(3.21 MB TIF)

Figure S3 TH-17 polarizing cytokines are preferentially induced by fungal hyphae in human DCs. IL-1b (A), IL-6 (B), and IL-12 (C) production by DCs stimulated with hyphae of Aspergillus (white bars) or Rhizopus (black bars), or b-glucan (scattered bars) or LPS (gray bars) with (+) or without (−) pre incubation for 1 h with an anti-dectin-1 blocking antibody (10 µg/ml). Data are expressed as mean ± SEM values for DCs derived from three different donors. *, P<0.001; **, P<0.05 paired Student's t test.
(4.87 MB TIF)

Figure S4 Aspergillus hyphae drive the expansion of IL-17 producing memory CD4+ T cells. Flow cytometry to determine the percentage of cells producing IL-17 and IFN-γ among memory CD4+ T cells primed for 5 d in plates coated with anti-CD3 and anti-CD28, in the presence of supernatants of unstimulated DCs, or supernatants of DCs stimulated with either Aspergillus spores, or hyphae (above plots), and re-stimulated for 5 h with PMA and Ionomycin. Data are expressed as mean ± SEM values for DCs derived from five different donors.
(4.54 MB TIF)

Figure S5 Polymyxin B has no effect in IL-6 production by DCs activated with b-glucan or fungal hyphae. IL-6 production by DCs stimulated with hyphae of Aspergillus (white bars) or b-glucan (black bars scattered bars) or LPS (gray bars) with or without pre incubation for 10 min with increasing concentrations of polymyxin B (PMX-B; 0, 10, 100 µg/ml). Data shown are representative of 2 independent experiments.
(5.19 MB TIF)
