# Supplementary figures and images for: Generation of IL-23 Producing Dendritic Cells (DCs) by Airborne Fungi Regulates Fungal Pathogenicity via the Induction of TH-17 Responses
Source: PLoS One. 2010 Sep 23;5(9):e12955. doi: 10.1371/journal.pone.0012955 (PMC2944889; doi:10.1371/journal.pone.0012955)

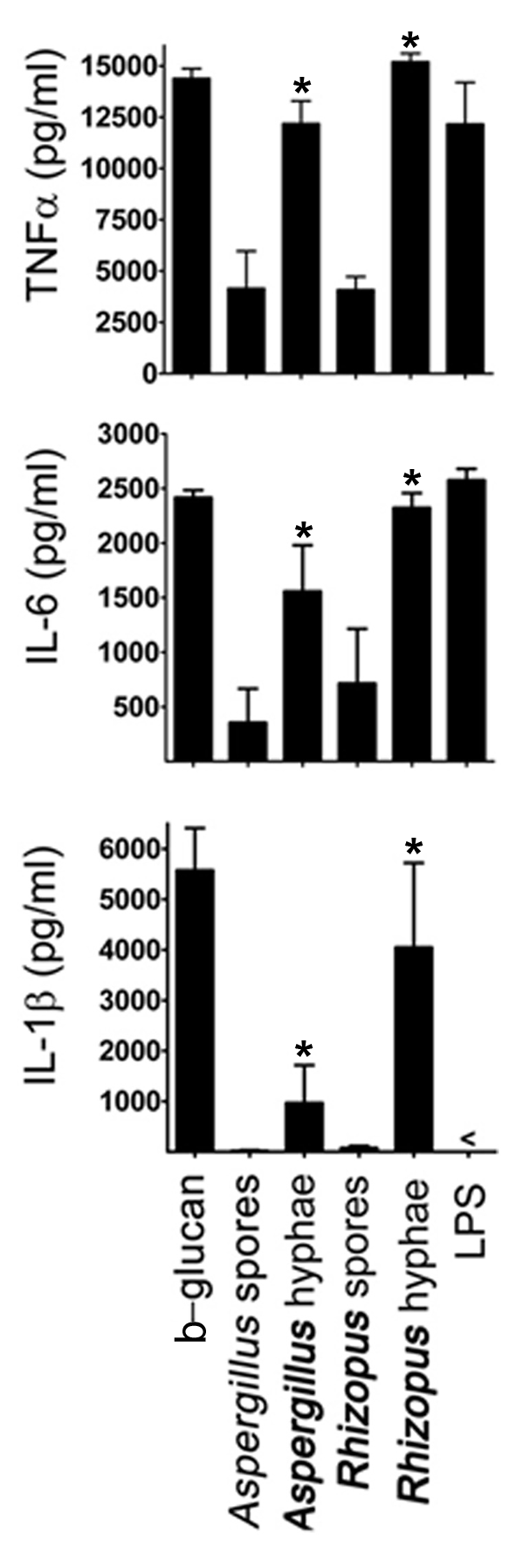

Supplement: Figure S1 — TH-17 polarizing cytokines are preferentially induced by fungal hyphae in human DCs. IL-1b (A) IL-6 (B) and TNF-a produced by human monocyte-derived DCs (1×10^6^ cells per ml) following overnight stimulation with purified b-glucan (curdlan, 100 µg/ml), resting (spores) or invasive (hyphae) stages of growth of each opportunistic fungus (Aspergillus and Rhizopus) at a 1∶1 ratio. Data are expressed as mean ± SEM values for DCs derived from three different donors. *, P<0.001, paired Student's t test. (1.08 MB TIF) [file pone.0012955.s001.tif]

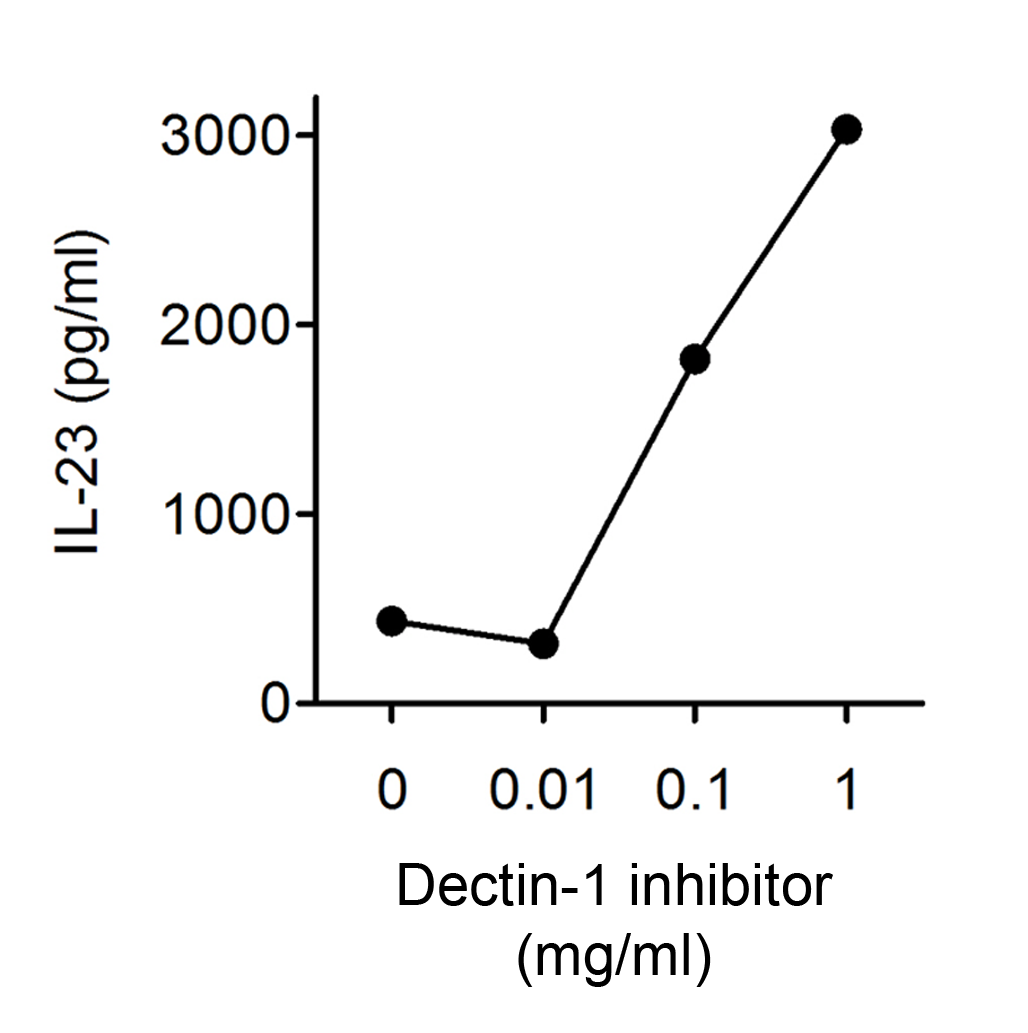

Supplement: Figure S2 — Dectin-1 inhibitor laminarin has no effect on DC activation by TLR ligands. IL-23 production by human monocyte-derived DCs pre incubated for 1 h with increasing concentrations of the dectin-1 inhibitor laminarin (0, 0.01, 0.1, and 1 mg/ml) and subsequently stimulated with LPS (100 ng/ml). Data shown are representative of 2 independent experiments. (3.21 MB TIF) [file pone.0012955.s002.tif]

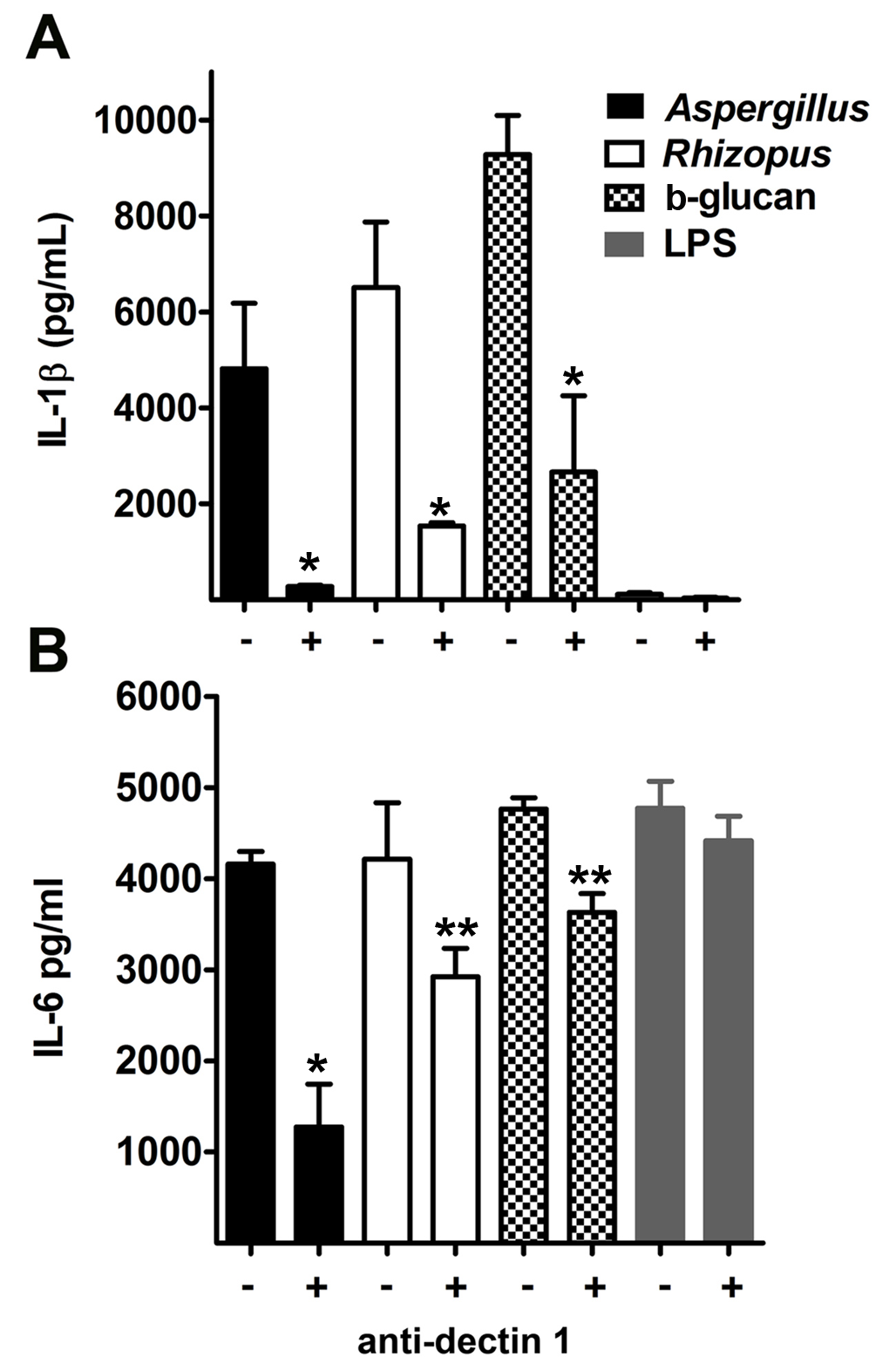

Supplement: Figure S3 — TH-17 polarizing cytokines are preferentially induced by fungal hyphae in human DCs. IL-1b (A), IL-6 (B), and IL-12 (C) production by DCs stimulated with hyphae of Aspergillus (white bars) or Rhizopus (black bars), or b-glucan (scattered bars) or LPS (gray bars) with (+) or without (−) pre incubation for 1 h with an anti-dectin-1 blocking antibody (10 µg/ml). Data are expressed as mean ± SEM values for DCs derived from three different donors. *, P<0.001; **, P<0.05 paired Student's t test. (4.87 MB TIF) [file pone.0012955.s003.tif]

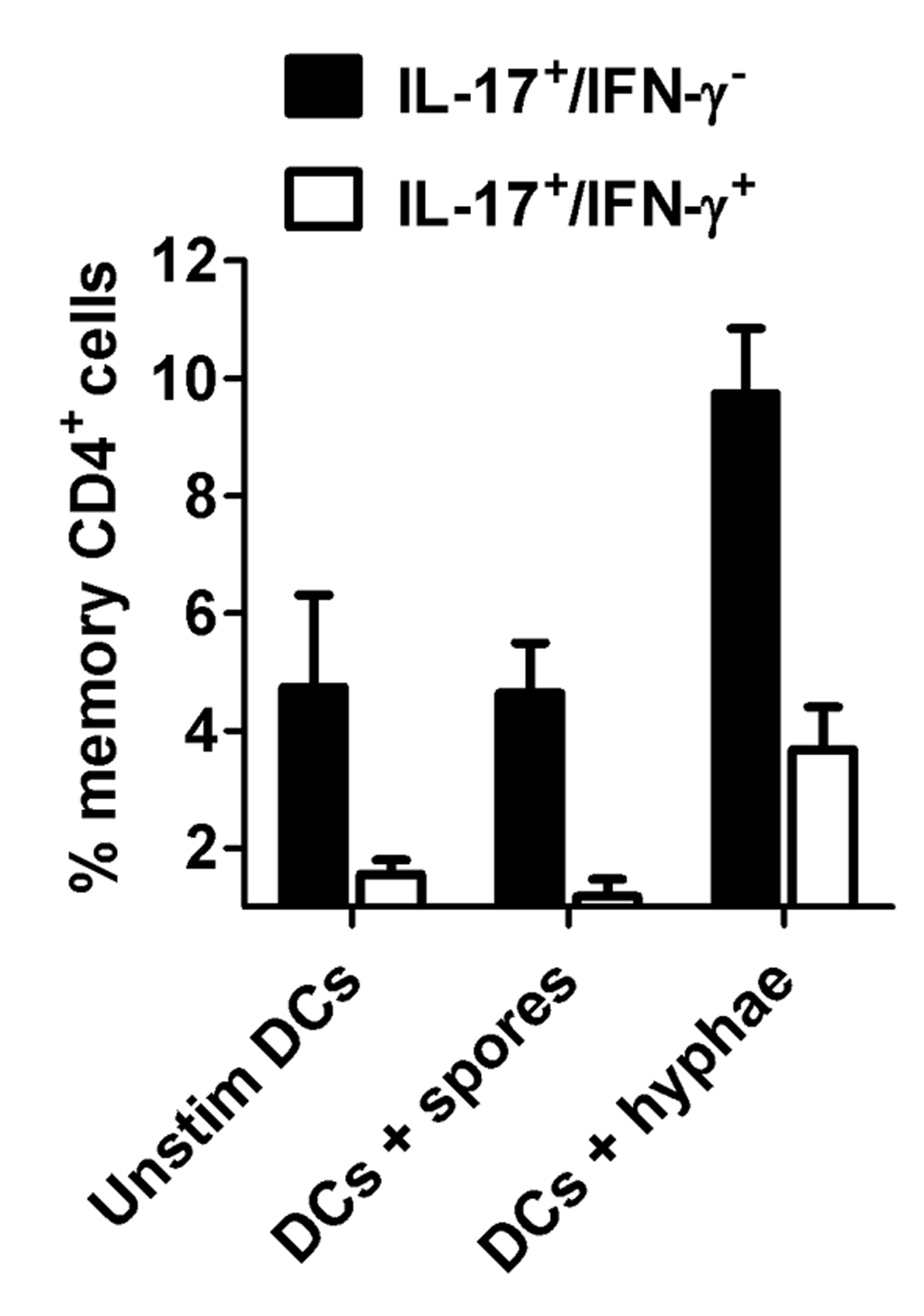

Supplement: Figure S4 — Aspergillus hyphae drive the expansion of IL-17 producing memory CD4+ T cells. Flow cytometry to determine the percentage of cells producing IL-17 and IFN-γ among memory CD4+ T cells primed for 5 d in plates coated with anti-CD3 and anti-CD28, in the presence of supernatants of unstimulated DCs, or supernatants of DCs stimulated with either Aspergillus spores, or hyphae (above plots), and re-stimulated for 5 h with PMA and Ionomycin. Data are expressed as mean ± SEM values for DCs derived from five different donors. (4.54 MB TIF) [file pone.0012955.s004.tif]

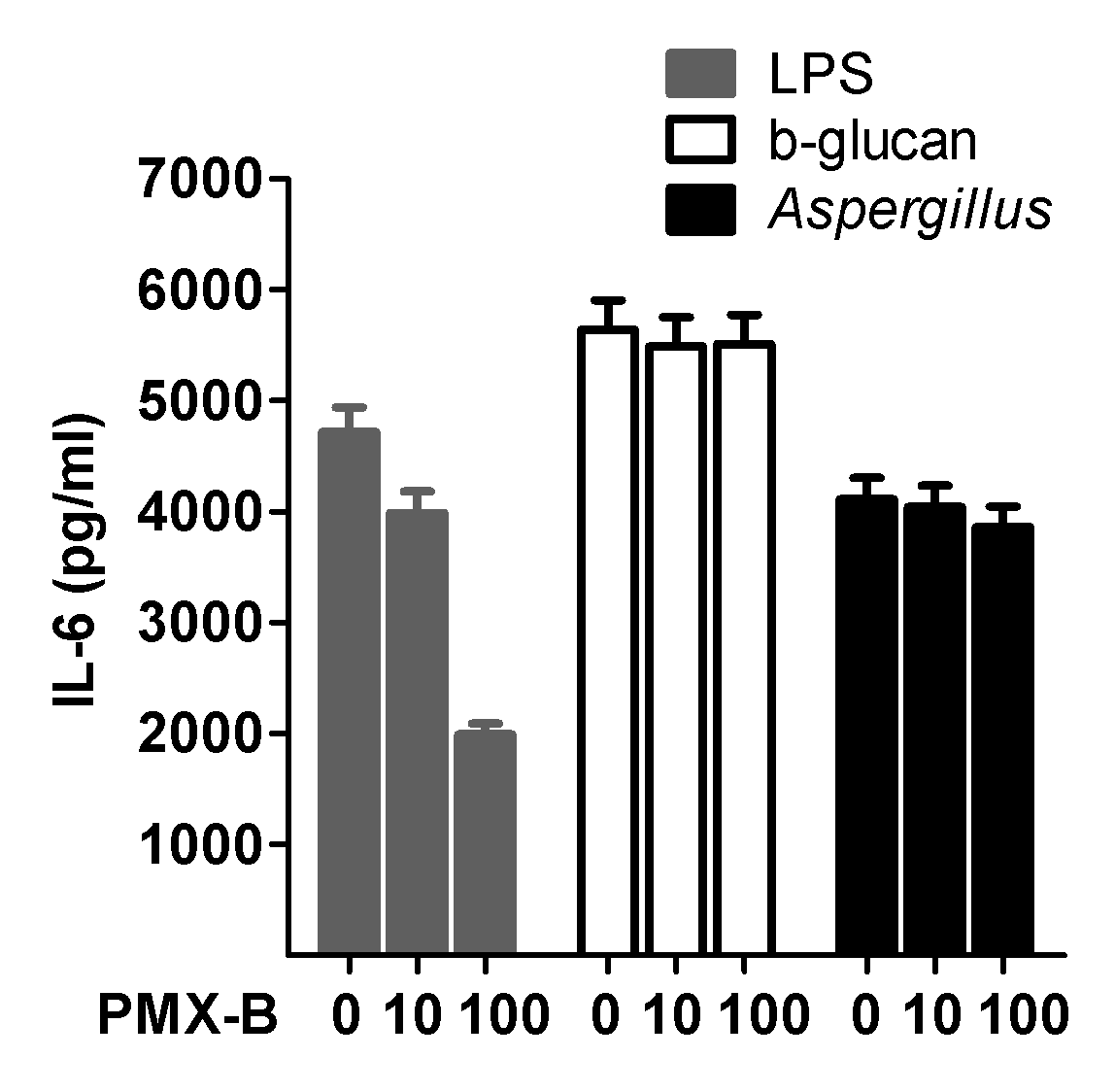

Supplement: Figure S5 — Polymyxin B has no effect in IL-6 production by DCs activated with b-glucan or fungal hyphae. IL-6 production by DCs stimulated with hyphae of Aspergillus (white bars) or b-glucan (black bars scattered bars) or LPS (gray bars) with or without pre incubation for 10 min with increasing concentrations of polymyxin B (PMX-B; 0, 10, 100 µg/ml). Data shown are representative of 2 independent experiments. (5.19 MB TIF) [file pone.0012955.s005.tif]
